# Supplementary material for: Spontaneous Development of Dental Dysplasia in Aged Parp-1 Knockout Mice
Source: Cells. 2019 Sep 27;8(10):1157. doi: 10.3390/cells8101157 (PMC6829344; doi:10.3390/cells8101157)
Supplement: Supplementary file 1 [file cells-08-01157-s001.pdf]

cells

MDPI

*Article*

# Spontaneous Development of Dental Dysplasia in Aged *Parp-1* Knockout Mice

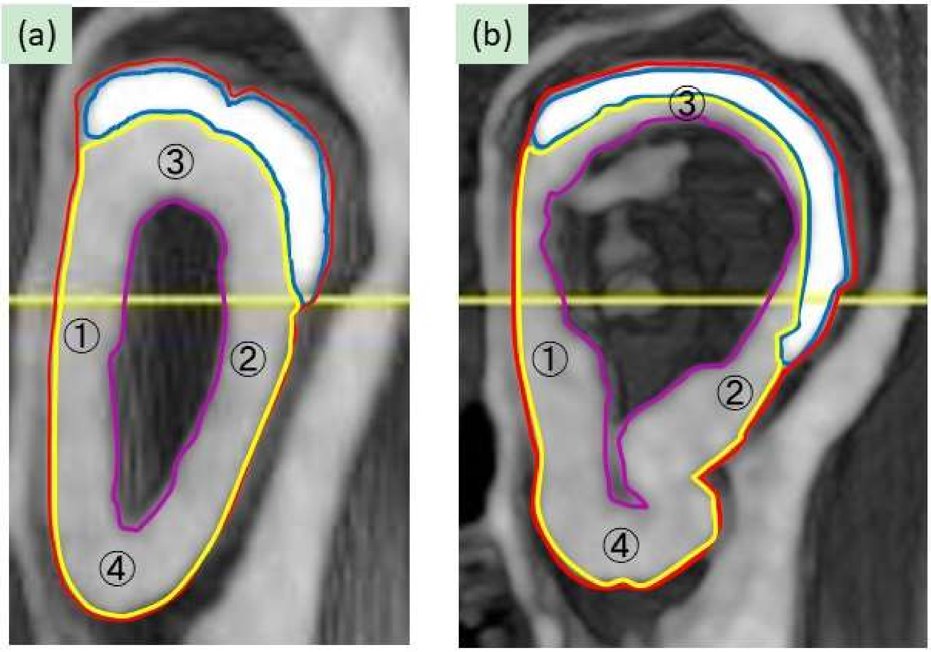


**Supplemental Figure 1.** Definition of areas of tooth elements. Representative CT images of incisors of aged Parp-1+/+ (a) and Parp-1-/- mice (b). The areas of enamel, dentin, pulp and the whole of the tooth were defined as encircled with blue, yellow, purple and red lines, respectively. The calcification level of dentin was defined as average of four points; ① medial, ②distal, ③ labial, and ④ palatal points of dentin.
